# Invasion-Related Factors as Potential Diagnostic and Therapeutic Targets in Oral Squamous Cell Carcinoma—A Review

**DOI:** 10.3390/ijms19051462

**Published:** 2018-05-14

**Authors:** Samadarani B. S. M. Siriwardena, Takaaki Tsunematsu, Guangying Qi, Naozumi Ishimaru, Yasusei Kudo

**Affiliations:** 1 Department of Oral Pathology, Faculty of Dental Sciences, University of Peradeniya, Peradeniya 20400, Sri Lanka; samadarani@yahoo.com; 2 Department of Pathology and Laboratory Medicine, Tokushima University Graduate School of Biomedical Sciences, Tokushima 770-8503, Japan; tsunematsu@tokushima-u.ac.jp; 3 Department of Pathology and Physiopathology, Guilin Medical University, Guilin 541004, China; qgy@glmc.edu.cn; 4 Department of Oral Molecular Pathology, Tokushima University Graduate School of Biomedical Sciences, Tokushima 770-8504, Japan; ishimaru.n@tokushima-u.ac.jp

**Keywords:** oral squamous cell carcinoma, invasion, metastasis, epithelial mesenchymal transition (EMT), cell adhesion, tumor microenvironment, cell signaling, microRNA

## Abstract

It is well recognized that the presence of cervical lymph node metastasis is the most important prognostic factor in oral squamous cell carcinoma (OSCC). In solid epithelial cancer, the first step during the process of metastasis is the invasion of cancer cells into the underlying stroma, breaching the basement membrane (BM)—the natural barrier between epithelium and the underlying extracellular matrix (ECM). The ability to invade and metastasize is a key hallmark of cancer progression, and the most complicated and least understood. These topics continue to be very active fields of cancer research. A number of processes, factors, and signaling pathways are involved in regulating invasion and metastasis. However, appropriate clinical trials for anti-cancer drugs targeting the invasion of OSCC are incomplete. In this review, we summarize the recent progress on invasion-related factors and emerging molecular determinants which can be used as potential for diagnostic and therapeutic targets in OSCC.

## 1. Introduction

According to the latest cancer statistics, oral squamous cell carcinoma (OSCC) is the leading cause of cancer related deaths in men, and it contributes to approximately 23% of deaths caused by all cancer types in men [1]. Cancerous metastasis is the most important prognostic factor of OSCC as in other carcinomas. Like most epithelial cancers, OSCC develops through the accumulation of genetic and epigenetic alterations in a multistep process. Recent molecular studies have advanced our understanding of the disease and provided a rationale to develop novel strategies for early detection, classification, prevention, and treatment. In the early step of metastasis, cancer cells acquire the reduction of cell-to-cell adhesion and mobility. Invasion is a highly dynamic process that involves a complex interplay between cell-intrinsic elements, and acquisition of invasive capabilities ultimately allows transmigration through the basement membrane (BM). The clinical significance of invasive properties is affected not only by the local region, but also by regional lymph node metastasis with extra capsular invasion. Depending on the primary site, OSCC cells can invade the underlying connective tissue as the first event. The pattern of invasion at the tumor invasive front within OSCC tissue, first described by Bryne et al. [2], is directly associated with the outcome of patients. When individual tumor cells are observed in the invasive front, the rate of lymph node metastasis greatly increases and prognosis worsens [3,4,5]. Depth of invasion in tongue tumors is also well associated with survival [6]. Indeed, when tumor thickness shows over 2 mm, nodal metastasis is frequently observed [6]. Therefore, elective neck dissection is recommended despite clinically node-negative [5]. Bone invasion is frequently observed in OSCC and it can be categorized as erosive and infiltrative. The latter type gives a 4-fold increased risk of death with disease. Furthermore, bone invasion is associated with more aggressive tumor spread, with high frequency of recurrence [7]. In addition, vascular and perineural invasion of OSCC cells are prognostic factors that give local recurrences, and regional and distant metastasis. A recent study showed that the presence of perineural invasion in tongue SCC predicted worse disease-specific survival, with distant recurrence as the most common pattern of failure [8]. The head and neck region is highly populated with rich neural networks. In particular, tongue and maxilla show rich vasculature.

In order to gain invasive properties, cancer cells require the molecular and genetic alterations in OSCC as in other types of cancer. In addition, the stromal compartments, including both stromal cells and extracellular matrices (ECMs), acquire these molecular and genetic changes during OSCC progression. Initiation and maintenance of the invasion process are mainly regulated by various signaling pathways. Understanding these genetic events eventually leads to better treatment, and thus, good prognostication. As a tumor progresses within the epithelium, the underlying stromal changes occur. The number of stromal cells, such as fibroblasts, macrophages, and pericytes, increases with tumor progression (9). Moreover, the stiffness of the ECM increases [9]. Subsequently, the communication between cancer cells and stromal cells via BM leads to the invasion by breaching BM. Previous reports provided the following mechanisms: (i) proteolytic degradation by matrix metalloproteinases (MMPs) and serine protease separase; (ii) mechanical forces; and (iii) reduced or abnormal synthesis of BM components around invasive cancers [10]. The following section describes the biology and mechanisms of invasion-related factors in OSCC and discusses these factors for prognostic and therapeutic targets.

## 2. Invasion-Related Cell Adhesion Molecules

**Figure 1:**
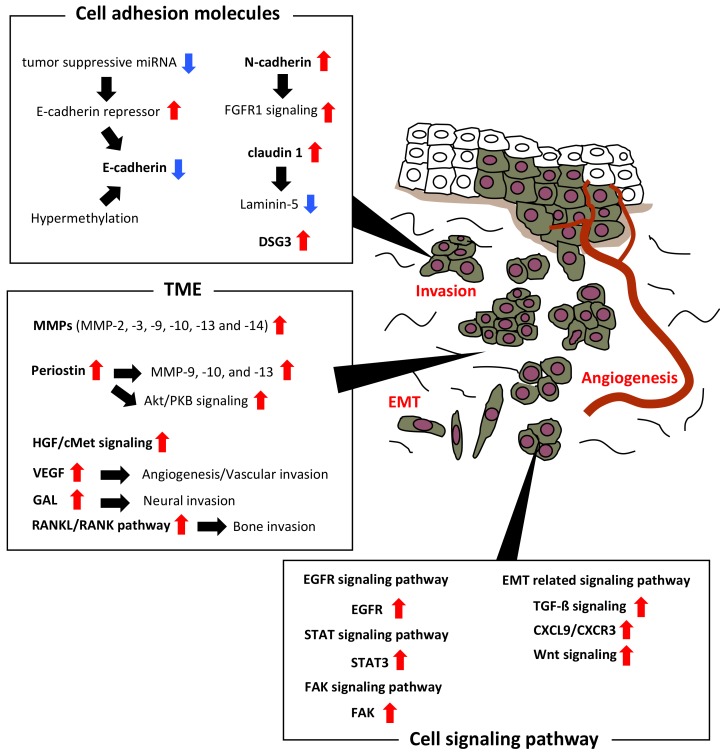
Invasion-related molecules in oral squamous cell carcinoma (OSCC). The figure shows invasion-related cell adhesion molecules and invasion-related molecules in a tumor microenvironment (TME), and a cell signaling pathway in OSCC. Red arrows show upregulation in OSCC. Blue arrows show downregulation in OSCC.

Abnormal regulation of cell adhesion molecules, (e.g., *E*-cadherin, Neural-cadherin (*N*-cadherin), claudin, and desmoglein (DSG)) is involved in the invasion of OSCC cells. The following section describes the abnormality of these invasion-related cell adhesion molecules in OSCC (Table 1 and Figure 1).

### 2.1. E-Cadherin

Cell–cell adhesion is mediated by *E*-cadherin. It is well known that reduction in *E*-cadherin stimulates the invasion of cancer cells. A meta-analysis indicates that a low level of *E*-cadherin is related to poor prognosis due to the phenotypic changes in increased motility and invasiveness of cancer cells [43]. Epithelial-mesenchymal transition (EMT) is a crucial process in cancer progression, providing cancer cells with the ability to escape from the primary focus to invade stromal tissues and to migrate to distant regions. EMT is the process by which epithelial cells lose epithelial phenotype and gain mesenchymal phenotype. In cancer tissue, EMT-caused cells increase the migratory capacity and degradation ability of extracellular matrix, then escape from the primary tumor and metastasize [44]. Downregulation of *E*-cadherin and upregulation of mesenchymal molecules are the hallmark of EMT. During EMT, cancer cells lose their cell-to-cell attachment by decreasing *E*-cadherin expression, due to hypermethylation of the promoter region or transcriptional repression caused by Zinc finger E-box-binding homeobox 1 (ZEB1), ZEB2, Snail, Slug (also known as SNAI2), and TWIST [45,46]. Several reports demonstrate that loss of *E*-cadherin expression in OSCC is induced by epigenetic mechanism. Hypermethylation of *CDH1* promoter region correlates with loss of *E*-cadherin expression in the most invasive and metastatic area of OSCC [11,12]. During EMT, the miR-200 family (miR-200a, -200b, -200c, -141, and -429) is shown as the most altered microRNA (miRNA) [47]. The miR-200 family makes a double negative feedback loop with ZEB1/ZEB2 to regulate cellular phenotype and maintains *E*-cadherin expression by directly suppressing ZEB1/ZEB2 [48,49]. In EMT-caused cells, the downregulation of miR-200 family induces expression of ZEB1/ZEB2, resulting in *E*-cadherin suppression [50]. To identify the invasion-related miRNAs, we previously compared the miRNA expression profiles between parent OSCC cells and their highly invasive clone [13]. We also identified miR-200 family as the downregulated miRNA in a highly invasive clone. In addition to miR-200 family, miR-203 is identified as the downregulated miRNA in a highly invasive clone. Reduced expression of miR-203 is involved in the invasion of OSCC cells via upregulation of NUAK family kinase 1(NUAK1) and SNAI2 [13]. A recent paper showed that cells expressing the partial EMT program, spatially localized to the leading edge of primary tumors in head and neck squamous cell carcinoma by single cell transcriptomic analysis [51]. Several reports demonstrated that that p-EMT program is distinct from full EMT programs derived from cell lines and tumor models, as well as from “mesenchymal” signatures derived from bulk tumor profiles [52,53]. Importantly, partial EMT is an independent predictor of nodal metastasis in head and neck squamous cell carcinoma [51]. Therefore, EMT and/or partial EMT-related molecules can be a prognostic marker in OSCC. As the detailed mechanism of EMT induction and partial EMT induction in OSCC is still unclear, further experiments will be required. It is known that the extracellular domain of *E*-cadherin can be proteolytically cleaved and released from the cell surface and can be detected in the circulation. The levels of soluble *E*-cadherin in the circulation reflect the progression of cancer and can be used as a diagnostic marker [54]. However, there are no studies on serum levels of *E*-cadherin in OSCC.

### 2.2. N-Cadherin

*N*-cadherin is an integral membrane, calcium-binding glycoprotein that mediates the intercellular adhesion of neuronal cells and other various types of non-neuronal cells [55]. The loss of *E*-cadherin and the gain of *N*-cadherin expression are known as the “cadherin switching” [56]. Cadherin switching is thought to reflect an EMT, whereby tumor cells are released from *E*-cadherin-dependent cell–cell interactions, and acquire a motile phenotype through the induction of *N*-cadherin. *N*-cadherin promotes invasive ability through activating Fibroblast growth factor receptor 1 (FGFR1) signaling by inhibiting FGFR1 internalization in breast cancer cells [57]. *N*-cadherin expression correlates with EMT phenotype and malignant behavior of OSCC [14]. Consistent with these findings, overexpression of FGFR1 correlates with EMT status with *N*-cadherin expression [15]. Interestingly, FGFR1-specific inhibitor PD173074 suppresses the invasion of OSCC cells [15]. *N*-cadherin can be a predictive marker for EMT induction and a prognostic marker in OSCC cells.

### 2.3. Claudin

Claudins are transmembrane proteins at the tight junction that create a seal between adjacent polarized epithelial cells. Claudins have two groups: namely classic and non-classic subgroups. Abnormal expression of claudin results in the structural and functional alterations at tight junctions which enhance the motility and invasion of cancer cells [58]. In particular, claudin-1 and -4 have been shown to be critical for the function of tight junctions [59]. Overexpression of claudin-1 is associated with local recurrence and poor survival via high probability of perineural and lymphatic invasion in OSCC [16]. Furthermore, claudin-1 knockdown decreases the invasion of OSCC cells [17]. Previous reports suggest that claudins may be involved in cancer progression through the complex interaction with several ECM elements. The inhibition of claudin-1 expression in OSCC cells diminished invasion and reduced degradation of laminin-5, an important component of the BM, via inactivation of MMP-2 and Membrane type 1-MMP (MT1-MMP) [17]. These findings indicate that claudin-1 appears to be a potential biomarker of the more progressive lesions and consequently poor clinical outcome of OSCC patients.

### 2.4. DSG

DSG glycoproteins (DSG1–4) are a group of cadherin in desmosomal intercellular junction that establishes a link between adjacent cells [60]. The desmosomes are known to play a role in malignant process. DSG3 is one of the component in the desmosome, and disorder of DSG3 is known to be related with pemphigus vulgaris via loss of cell-to-cell adhesion by autoantibodies against DSG3 [61]. Although previous reports show that downregulation of DSG3 is observed in OSCC and breast cancer [18,62], a large-scale microarray study by Chung et al. [63] using 60 OSCC samples revealed that a subtype of tumors contained genes involved in the function of desmosome including DSG3 are overexpressed in poor outcome patients. Moreover, DSG3 was identified as a highly-expressed molecule in OSCC by differential display analysis to compare the gene expression profiles between OSCC and normal epithelial tissues [64]. Indeed, DSG3 knockdown suppresses tumor growth and metastasis of OSCC cells in vivo [64]. Interestingly, overexpression of DSG3 enhances membrane protrusions, and cell spreading and rounding that are the necessary prerequisites for cell migration/invasion [64,65]. Importantly, research evidence suggests that DSG3 platforms which can identify positive and negative nodes can be achieved within an intra operative timeframe, which ultimately reduces unnecessary lymph node resection [19,66]. DSG3 can be a potential diagnostic and therapeutic target in OSCC.

## 3. Invasion-Related Molecules in Tumor Microenvironment (TME)

Under normal circumstances, cells migrate during embryonic development and settle in a distant location via EMT and mesenchymal-epithelial transition (MET). EMT allows polarized epithelial cells to acquire mesenchymal cell phenotype having multiple biochemical changes which enhance migratory capacity, invasiveness, elevated resistance to apoptosis, and greatly increased production of ECM components [67]. Tumor cells mediate proteolytic digestion of ECM components termed as invadopodia is essential to the invasive process. Collectively, ECM components surrounded in cancer cells are known as TME. Substantial evidence indicates that intratumoral heterogeneity among malignant and non-malignant cells, and their interactions within the TME, are critical to diverse aspects of tumor biology [68,69]. The molecules in TME, such as MMP, periostin, hepatocyte growth factor (HGF), vascular endothelial growth factor (VEGF), and galanin (GAL), promote invasion of OSCC cells. The following section describes these invasion-related molecules in TME (Table 1 and Figure 1).

### 3.1. Matrix Metalloproteinases (MMPs)

Degradation of restrictive ECM proteins is mediated by the action of MMPs. ECM degradation by MMPs plays a pivotal role in cancer progression by promoting motility, invasion, and angiogenesis. Many studies have shown that MMP expression is increased in OSCCs. Previous our review summarize the current knowledge of MMPs, specifically MMP-1, -3, -7 -10, -12, -13, 14, and -19, that are highly expressed in OSCCs and involved cancer invasion and angiogenesis [20]. Among MMPs, MMP-2, -9, and -14 are associated with invadopodia [70]. MT1-MMP (also known as MMP-14) is considered a central factor of invadopodia-mediated ECM degradation. Furthermore, MT1-MMP is directly regulated by Src kinase via phosphorylation on Tyr573 and activates MMP-2, -3, and -9 [21]. Phosphorylation of MT1-MMP on Tyr573 has been shown to be required for tumor growth and invasion both in vitro and in vivo. Hence, trafficking of MT1-MMP on the cell surface is involved in the cancer invasion. Expression of MT1-MMP is directly associated with metastasis and poor prognosis in OSCC [21]. Although clinical trials fail when MMP activity is blocked, new therapeutic strategies aiming to target the specific MMPs have been proposed. Devy et al. [71] indicate that a monoclonal antibody, DX-2400 against the catalytic domain of MT1-MMP, suppresses angiogenesis, tumor formation, and metastasis via blocking MMP2 cleavage in tumor and endothelial cells. The above drug or blocking substrates were successful in preclinical studies [72], and this could be a promising potential therapeutic target in the future. A murine monoclonal antibody REGA-3G12 against the catalytic domain of MMP-9 specifically inhibits MMP-9 activity [73]. MMP-9 is secreted by various human cancer cells and can be secreted by infiltrating immune cells including macrophages and neutrophils. MMP-9 is known to contribute to tumor progression including angiogenesis and invasion. Therefore, REGA-3G12 may be an effective cancer therapeutic drug. However, to date, clinical trials of this drug not yet have been initiated. In our previous study, cancer invasion-related factors were identified by comparing the gene expression profiles between parent and highly invasive clone of cancer cells [74]. MMP-13 is identified as a common upregulated gene by cancer invasion-related factors [75]. Although MMP-13 slightly promoted tumor invasion, MMP-13 is involved in tumor angiogenesis via activation of focal adhesion kinase (FAK) and extracellular signal-regulated kinase (ERK). Thus, elevated production of MMPs in TME contributes to tumor invasion. Inhibition of the function of MMPs by drugs including blocking antibody and blocking cleavage may be an effective for tumor progression in OSCC.

### 3.2. Periostin

Periostin is known as a component of ECM and is overexpressed in various cancers including OSCC [76]. In OSCC, periostin promotes tumor angiogenesis, migration, and metastases [22], and its overexpression has been shown to enhance invasion and anchorage-independent growth and spread [76]. Overexpression of periostin promotes invasion and metastasis by activation of Akt/protein kinase B (PKB) signaling via αvβ3 integrin [77]. Thus, periostin is believed to play a role during invasion, angiogenesis, and metastasis, as demonstrated by in vitro and in vivo experiments [23,78]. Recent finding suggests that periostin may have a role in sprouting neovascular endothelial tips of disseminated tumor cells, promoting breast cancer cell outgrowth in a tumor-suppressive microenvironment [79]. Periostin is a driver of the EMT and induces expression of MMP-9, MMP-10, and MMP-13, resulting in the degradation of ECM, believed to be crucial for local tumor spread and/or metastasis via invasion and neovascularization [24,25,75]. Furthermore, it is involved in remodeling the tumor microenvironment by promoting tumor survival, growth, and invasiveness [76]. Periostin-overexpressing human mammary epithelial cells acquire part of the multi-lineage differentiation potentials of mesenchymal stem cells and promote tumor growth and metastasis of human breast cancer cell line [80]. These data indicate that periostin is a critical matricellular component in remodeling tissue microenvironment in tumor growth and metastasis. Interestingly, the neutralizing antibody to periostin, MZ-1, suppressed tumor metastasis of periostin overexpressing ovarian cancer cell line by intra-peritoneal injection [81]. Furthermore, targeting periostin with a modified DNA aptamer, PNDA-3, that is capable of binding to periostin with high affinity and inhibiting its function markedly antagonized adhesion, migration, and invasion of breast cancer cells both in vitro and in vivo [82]. These findings suggest that periostin can be a potential therapeutic target for OSCC.

### 3.3. Hepatocyte Growth Factor (HGF)

Overexpression of HGF and its receptor c-Met have been reported in the majority of OSCCs [26]. Activation of HGF/c-Met pathway promotes EMT induction and has emerged as a potential therapeutic target. HGF is secreted by tumor associated fibroblasts within TME as an inactive proenzyme, and once cleavage occurs it become a heterodimer that is capable of binding to c-Met. This activates downstream signaling via adaptor molecules (i.e., growth-factor-receptor-bound protein 2 (Grb2) and Grb2-associated binder 1 (Gab1)) ultimately promoting invasion/proliferation and cell survival [27]. Aberrant HGF/c-Met signaling in OSCC promotes tumor progression by increasing the invasive capacity by acquiring an elongated spindle-like morphology [26]. Several agents have been developed to target HGF/c-Met and its downstream molecules such as tyrosine kinase inhibitors (TKIs), monoclonal antibodies, and competitive HGF antagonists and c-Met receptor decoys. Crizotinib (PF-2341066)—an orally available small-molecule inhibitor of c-Met—exhibits cyto-reductive antitumor efficacy through anti-proliferative and antiangiogenic mechanisms [83]. Crizotinib significantly inhibits tumor proliferation and abrogation of downstream AKT signaling, and reduces blood vessel density in vivo [28]. Furthermore, increased expression of c-Met correlated with resistance to platinum-based agents, radiation, and to epidermal growth factor receptor (EGFR)-targeting agents in OSCC [84]. Although there were several monoclonal antibodies against HGF, to date ficlatuzumab remains the only antibody against HGF undergoing clinical evaluation in OSCC [84].

### 3.4. Vascular Endothelial Growth Factor (VEGF)

Most solid tumors induce neoangiogenesis by producing angiogenic factors for the tumor cells’ nourishment. In the metastatic cascade, the first step is invasion followed by intravasation and extravasation. The most common route of metastasis in OSCC is via lymphatics, and traditionally lymphatic invasion is a passive process. However, recent data suggest that lymphangiogenesis in the tumor site may provide more opportunities for cancer intravasation. However, further studies are necessary in order to assign it as a therapeutic target. In contrast, vascular invasion is an active process. There are a number of factors that have been demonstrated to enhance angiogenesis such as VEGF and HGF. VEGF is one of the best known and well established regulators of angiogenesis to date. Therefore, specific targeting of VEGF signaling has been one of the key avenues in developing anti-angiogenic therapies. VEGF neutralizing antibody, Bevacizumab (also known as Avastin), has been approved for use in a variety of cancer types, such as lung cancer and colon cancer [85]. In OSCC, VEGF overexpression is frequently observed [29]. Indeed, a number of clinical trials have examined the combinatorial therapeutic effects of bevacizumab with other drugs for the treatment of recurrent or metastatic OSCC [30].

### 3.5. Galanin (GAL)

Some head and neck tumors exhibit a tendency towards neural invasion, and perineural invasion predicts poor survival in OSCC. Perineural invasion, as well as lymphovascular invasion, are important processes of metastasis in OSCC. Therefore, inhibition of perineural invasion can be an important strategy for OSCC treatment. Recent studies revealed that cancer cells have an innate ability to actively migrate along axons and is supported by various cell types in the perineural niche that secrete multiple growth factors and chemokines. Neuropeptide GAL initiates nerve-tumor crosstalk via activation of its G protein-coupled receptor, GALR2. Prostaglandin E2 promotes cancer invasion, and in a feedback mechanism, GAL released by cancer induces neuritogenesis, facilitating perineural invasion. Therefore, GALR2-induced pathway is a potential treatment target of perineural invasion [31].

## 4. Invasion-Related Molecules in Cell Signaling Pathway

Cell signaling is part of any communication process that governs basic activities of cells and coordinates all cell actions. The cell signaling pathway, such as receptor activator of nuclear factor-κB ligand (RANKL)/RANK, EGFR, signal transducer and activator of transcription (STAT), and focal adhesion kinase (FAK) are involved in the invasion of OSCC cells. As described above, EMT is an important process of OSCC progression. The following section describes the invasion-related molecules in cell signaling pathway and EMT related signaling pathway (Table 1 and Figure 1).

### 4.1. Receptor Activator of Nuclear Factor-κB Ligand (RANKL/RANK)

OSCC readily invades the proximal jaw bone and this is closely associated with poor prognosis. Osteoclastogenesis is regulated by a complex signaling system that involves three essential molecules: RANKL, its receptor (RANK), and its decoy receptor osteoprotegerin (OPG). Recently, Chuang et al. [32] compared RANKL expression between buccal SCC without bone invasion (25 cases) and gingival SCC with invasion (15 cases) and showed no difference: however, the buccal SCC cells do possess the potential to induce osteoclastogenesis through the RANKL/RANK pathway if triggered under appropriate conditions. Molecular control of RANKL gene expression in cancer cells is pivotal to our understanding of cancer progression. Jimi et al. [33] conclude that the inhibition of osteoclast differentiation and function by blocking RANKL/RANK constitutes with soluble RANK or OPG successfully prevents the development of bone invasion. A phase I study testing recombinant OPG in patients with multiple myeloma, or breast carcinoma-related bone metastases, is currently in progress [86], and there have not been any side-effects so far when administered as a single subcutaneous injection to patients [87]. A new humanized monoclonal anti-IL-6 antibody, MEDI5117, showed an inhibitory effect on cancer stem cells in OSCC and is currently in clinical trials for rheumatoid arthritis [88]. Another group demonstrated that RANKL expression is autoregulated via transcription factor NAFTc2 in OSCC cells, and treatment with OPG inhibited the autoregulation [34]. Hence, targeting molecules involving autoregulation of RANKL may be useful targets in controlling tumor growth and bone invasion.

### 4.2. Epidermal Growth Factor Receptor (EGFR)

Overexpression of the EGFR is frequently observed in OSCC, and increased activity in EGF signaling pathways has been associated with resistance to treatment and poor clinical outcome [35,89,90]. The EGFR is a transmembrane protein that is a receptor for members of the EGF family of extracellular protein ligands, such as EGF and transforming growth factor alpha (TGF-α) [91]. The EGFR binding with specific ligands activates intracellular signaling pathways that control growth, differentiation, survival and invasion [36,91,92]. The EGFR is therapeutically targeted by agents, such as a chimeric anti-EGFR monoclonal antibody (i.e., cetuximab, zalutumumab, nimotuzumab, panitumumab, MEHD7945A, necitumumab, and RO5083945), the multi-targeted small molecule tyrosine kinase inhibitors (TKI) (i.e., lapatinib, dacomitinib, afatinib, vandetanib, icotinib, and CUDC-101), and the anti-EGFR TKI (i.e., erlotinib) [37]. The chimeric anti-EGFR monoclonal antibody (mAb) cetuximab was the first molecularly targeted therapy to receive US Food and Drug Administration (FDA) approval for the treatment of OSCC.

### 4.3. Signal Transducer and Activator of Transcription (STAT)

Proteins of the STAT family mediate cellular responses to cytokines and growth factors. STAT3 is known to regulate expression of essential components of invasion and metastasis in various cancers including OSCC. Upstream receptors of STAT include IL-6, receptor tyrosine kinases (RTKs), vascular endothelial growth factor receptor (VEGFR), EGFR, Janus-activated kinases (JAK), and Src family kinases (SFK). Activated STAT3 up regulates the transcription of cyclin D1, survivin, and Bcl-XL [93]. Therapeutic agents targeting upstream receptors of STAT3, STAT3 domain, STAT3-DNA binding, and STAT3 transcription are still ongoing in early phase clinical trials in different stages [38,94].

### 4.4. Focal Adhesion Kinase (FAK)

FAK has been proposed as a new candidate for molecular-based therapeutic approaches. FAK is a multifunctional regulator of cell signaling within the TME [95]. FAK functions as a major mediator of signal transduction by cell surface receptors including integrins, growth factor, and cytokine receptors. Therefore, FAK plays a crucial role in carcinogenesis, especially in cell proliferation, cell motility, invasion, inhibition of apoptosis, angiogenesis, and immunosuppression. Increased levels of FAK mRNA in OSCC are correlated with tumor invasion and progression. Furthermore, overexpression of FAK is linked with poor survival in esophageal cancer and OSCC patients [39]. In TME, FAK favors tumor progression via the regulation of signaling pathways of endothelial cells, hematopoietic cells, platelets, macrophages, and fibroblasts. FAK activity promotes migration, proliferation, and survival of endothelial cells and stimulates tumor angiogenesis. FAK-mediated regulation of endothelial cell permeability can influence tumor metastasis [96]. Although not tested in OSCC, as a small molecule, FAK inhibitors are emerging as promising chemotherapeutics and combined treatment with FAK and SRC inhibitors demonstrated enhanced anti-tumor activity in small cell lung cancer [97]. So far, there are two clinical trials (Pfizer and GSK), and both trials found that the compounds are tolerated with low adverse events. Notably, in the Pfizer trial, some patients exhibited stable disease [96,98].

### 4.5. EMT Related Signaling Pathways

EMT related signaling pathways play a crucial role in tumorigenesis, mainly P13K/Akt signaling pathway and its partners transforming growth factor-ß (TGF-β), NF-κB, Ras, and Wnt/β-Catenin pathways. Recent study shows that the chemokine C-X-C motif chemokine ligand 9 (CXCL9)/receptor CXCR3 axis induces EMT and cytoskeleton rearrangement via activation of Akt signaling pathway in OSCC [40]. Indeed, overexpression of CXCL9/CXCR3 promotes migration and invasion of OSCC cells [40]. It is well known that TGF-β is a key initiator of EMT, which can induce artificial EMT of normal epithelial cells, as well as cancer cells [99,100]. TGF-β upregulates expression of key EMT regulators, including Snail and δEF1/SIP1, in epithelial and cancer cells [41]. Several intracellular signals, such as tumor necrosis factor-α, FGF-2, FGF-4, EGF, and HGF enhance TGF-β signaling to promote tumor invasion/metastasis and EMT [101,102]. In OSCC, TGF-β not only induces EMT to increase the capacity for invasion, but also promotes factors which prolong osteoclast survival [42]. As EMT is involved in malignant behaviors of cancer cells, inhibition of these EMT-related signal transduction pathways can be used as new tool in anticancer therapy. For example, Akt inhibitor redford can inhibit a combination of P13K and Akt which is in Phase II clinical trials [103]. Sulindac inhibits Wnt signaling pathway via downregulating ß-Catenin and Cyclin D1 [104]. Jang et al. [105] reported that inhibiting Wnt pathway by low-density lipoprotein receptor-related protein 6 (LRP6) reversed the EMT restoring the epithelial phenotype. Further, by blocking the Hedgehog signaling pathway with CUR0199691, it significantly weakened the ability of invasion in breast cancer [106].

## 5. Invasion-Related miRNAs

MicroRNAs are a class of highly-conserved 18–25 nucleotide, small, non-cording RNAs, which regulate a number of gene expressions through translational repression or mRNA degradation. They have important roles not only in various biological processes including cell proliferation, stress resistance, and metabolism, but also in pathogenesis. Many reports have shown that several miRNAs have oncogenic or tumor suppressive activities [107,108]. Here we listed various oncogenic or tumor suppressive miRNAs that are involved in the invasion and EMT induction in OSCC (Table 2). EMT-inducing molecules are upregulated by downregulated EMT-related miRNAs in OSCC. Invasion-suppressive molecules are downregulated by upregulation of oncogenic miRNAs in OSCC, and invasion-promoting molecules are upregulated in OSCC by downregulated tumor suppressive miRNAs in OSCC. These miRNAs can be a therapeutic modality and a diagnostic marker for targeting invasion of OSCC cells.

### 5.1. EMT-Related miRNAs

During EMT, cells lose adhesion and increase in motility by repression of *E*-cadherin expression, which also occurs during the initial stages of metastasis. As mentioned above, the miR-200 family and miR-203 is involved in EMT induction in OSCC [13,47,48,49,50]. The miR-200 family is believed to play an essential role in tumor suppression by inhibiting EMT. The miR-200 family makes a double negative feedback loop with ZEB1/ZEB2 to regulate cellular phenotype and maintains *E*-cadherin expression by directly suppressing ZEB1/ZEB2 [48,49]. In EMT caused cells, the downregulation of the miR-200 family induce expression of ZEB1/ZEB2, resulting in *E*-cadherin suppression [50]. miR-200 targets the *E*-cadherin transcriptional repressors ZEB1 and ZEB2. Indeed, knockdown of miR-141 and miR200b has been shown to reduce *E*-cadherin expression, thus increasing cell motility and inducing EMT [47,48]. miR-203 is also involved in EMT and invasion via targeting SNAI2 and NUAK1 [13]. Both miR-200 family and miR-203 are downregulated in OSCC cells with high invasive ability [13]. Moreover, several miRNAs, such as miR-153 (via targeting SNAI1 and ZEB2), miR-101 (via targeting enhancer of zeste homolog 2: EZH2) miR-27a-3p (via targeting yes-associated protein 1: YAP1), and miR-485-5p (via targeting p21 RAC1 activated kinase 1: PAK1) are involved in EMT induction [109,110,111,112].

### 5.2. Invasion-Related Oncogenic miRNAs

The following miRNAs are involved in the invasion of OSCC via targeting various genes. In OSCC, miR-21 (via targeting Dickkopf-related protein: DKK2), miR-29a (via upregulating MMP2), miR-196 (via targeting non-metastatic cells 4: NME4), miR-155 (via targeting B-cell CLL/lymphoma 6: BCL6), miR-24 (via targeting F-box and WD-40 domain protein 7: FBXW7), and miR-1275 (via upregulating Insulin-like growth factor 1 receptor: IGF-1R and C-C chemokine receptor type 7: CCR7) promote the invasion as an oncogenic miRNA [113,115,116,117,118,119]. Oncogenic miRNAs are frequently overexpressed in OSCC. Interestingly, oncogenic miRNAs are included in exosome from OSCC cells [120,144]. MiRNA array analysis identified two oncogenic miRNAs, miR-342-3p and miR-1246, that were highly expressed in exosomes isolated from a highly metastatic human OSCC cell line [120]. Exosomes, which are packed with RNA and proteins and are released in all biological fluids, are emerging as an important mediator of intercellular communication. To detect certain miRNAs can be used for useful tool for early detection and prediction of metastasis in OSCC.

### 5.3. Invasion-Related Tumor Suppressive miRNAs

On the other hand, tumor suppressive miRNAs inhibit the invasion of OSCC. In OSCC, tumor suppressive miRNAs are frequently downregulated. In OSCC, many tumor suppressive miRNAs are involved in invasion, as the following; miR-222 (via targeting MMP1 and manganese superoxide dismutase 2: SOD2), miR-138, miR-363 (via targeting podoplanin), miR-491-5p (via targeting G-protein-coupled receptor kinase-interacting protein 1: GIT1), miR-140-5p, miR-133b, miR-29b (via targeting Sp1), miR-125a (via targeting estrogen-related receptor α: ESRRA), miR-34a (via targeting MMP9 and MMP14), miR-329 and miR-410 (via targeting Wnt-7b), miR-143 (via targeting CD44 v3), miR-222 (via targeting ATP-binding cassette sub-family G member 2: ABCG2), miR-188 (via targeting SIX1), miR-196b, miR-23b and miR-27b (via targeting receptor tyrosine kinase MET), miR-200c-3p (via targeting chromodomain-helicase-DNA-binding protein 9: CHD9 and Werner syndrome ATP-dependent helicase, WRN), miR-205-p (via targeting the tissue inhibitor of metalloproteinases‑2: TIMP‑2), miR-22 (via targeting NLR family pyrin domain containing three: NLRP3), miR-195-5p (via targeting tripartite motif-containing protein: TRIM14), miR-30a-5p (via targeting fibroblast activation protein α: FAP), miR-376c-3p (via targeting HOXB7), miR-143 (via targeting hexokinase 2), miR‑375 (via targeting platelet‑derived growth factor‑A: PDGF-A), and miR-320a suppress the invasion as a tumor suppressive miRNA [114,121,122,123,124,125,126,127,128,129,130,131,132,133,134,135,136,137,138,139,140,141,142,143,145].

## 6. Conclusions

In this paper, we introduce several invasion-related cell adhesion molecules, invasion-related in TME, invasion-related molecules in cell signaling pathway, and invasion-related miRNAs (Table 1 and Table 2, and Figure 1). So far, there are numerous reports on invasion-related molecules in OSCC. However, the full scope of this mechanism has not yet been clarified. Among various molecules, we need to find out which factors can be critical targets for OSCC treatment through inhibiting invasion and metastasis. Moreover, various oncogenic and tumor suppressive miRNAs are involved in invasion of OSCC via targeting variety of genes. However, mutual relationships among various miRNAs and/or invasion-related molecules needs to be clarified. Our desire is to develop effective diagnostic and/or therapeutic targets against invasion and metastasis in OSCC.

## Figures and Tables

**Table 1:** Invasion-related molecules in OSCC.

Gene Name		Aberrant Expression in OSCC	Specific Function in OSCC	References
*E*-cadherin	Cell adhesion molecule	Downregulation hypermethylation	Acquisition of EMT phenotype including promoting invasion	[11,12,13]
*N*-cadherin	Cell adhesion molecule	Upregulation	Promote invasiveness via activating FGFR1 signaling pathway	[14,15]
Claudin-1	Cell adhesion molecule	Upregulation	Promoting invasion via structural and functional alterations of tight junctions	[16,17]
Desmoglein-3	Cell adhesion molecule	Downregulation Upregulation	Involved in desmosomal intercellular junction	[18,19]
MT1-MMP (MMP-14)	Matrix metalloprotease	Upregulation	Promoting invasion via degradation of ECM (Collagens I, II, and III; gelatins; aggrecan; fibronectin; laminin, fibrin)	[20,21]
MMP-2	Matrix metalloprotease	Upregulation	Promoting invasion via degradation of ECM (gelatins; VII, X and, XI; fibronectin; laminin; elastin; aggrecan)	[20]
MMP-9	Matrix metalloprotease	Upregulation	Promoting invasion via degradation of ECM (gelatins; collagens III, IV, and, V; aggrecan; elastin; entactin; vitronectin; *N*-telopeptide of collagen I)	[20]
Periostin	Component of ECM	Upregulation	Promoting angiogenesis, lymphangiogenesis, migration, and invasion	[22,23,24,25]
HGF	Growth factor	Upregulation	Promoting EMT induction via HGF/c-Met signaling	[26,27]
c-Met	Receptor	Upregulation	Promoting EMT, proliferation, and angiogenesis induction via HGF/c-Met signaling	[26,27,28]
VEGF	Growth factor	Upregulation	Angiogenesis	[29,30]
GAL	Neuropeptide	Downregulation	Perineural invasion	[31]
RANKL	Membrane protein	Upregulation	Bone invasion via induction of osteoclastogenesis	[32,33,34]
EGFR	Receptor	Upregulation	Activating P13K and Akt pathways	[35,36,37]
STAT3	Activator of transduction	Signal activation	Activating gene transcription involved in the essential components of invasion and metastasis	[38]
FAK	Mediator of signal transduction	Upregulation	Promoting invasion as a mediator of integrin and growth factors signaling	[39]
CXCL9	Chemokine	Upregulation	EMT induction and cytoskeleton rearrangement via activation of Akt signaling pathway	[40]
CXCR3	Chemokine receptor	Upregulation	EMT induction and cytoskeleton rearrangement via activation of Akt signaling pathway	[40]
TGF-β	Growth factor	Signal activation	EMT induction	[41,42]

**Table 2:** Invasion-related microRNA (miRNA) in OSCC.

Function	miRNA	Target Gene Etc.	References
EMT-related miRNAs	miR-200 family (miR-200a, miR-200b, miR-200c, miR-141, miR-429)	*ZEB1/ZEB2*	[13]
	miR-203	*SNAI2/NUAK1*	[13]
	miR-485-5p	*PAK1*	[109]
	miR-27a-3p	*YAP1*	[110]
	miR-101	*EZH2*	[111]
	miR-153	*SNAI1/ZEB2*	[112]
Oncogenic miRNAs	miR-21	*DKK2*	[113,114]
	miR-29a	upregulating MMP2	[115]
	miR-196	*NME4*	[116]
	miR-155	*BCL6*	[117]
	miR-24	*FBXW7*	[118]
	miR-1275	upregulating IGF-1R/CCR7	[119]
	miR-342-3p	included in exosome	[120]
	miR-1246	included in exosome	[120]
Tumor suppressive miRNAs	miR-222	*MP1/SOD2*	[114]
	miR-138	-	[121]
	miR-363	*podoplanin*	[122]
	miR-491-5p	*GIT1*	[123]
	miR-140-5p	-	[124]
	miR-133b	-	[125]
	miR-29b	*SP1*	[126]
	miR-125a	*ESRRA*	[127]
	miR-34a	*MMP9/MMP14*	[128]
	miR-329	*Wnt-7b*	[129]
	miR-410	*Wnt-7b*	[129]
	miR-143	*CD44v3/hrxokinase 2*	[130,131]
	miR-222	*ABCG2*	[132]
	miR-188	*SIX1*	[133]
	miR-196b	-	[134]
	miR-23b	*MET*	[135]
	miR-27b	*MET*	[135]
	miR-200c-3p	*CHD9/WRN*	[136]
	miR-205-5p	*TIMP-2*	[137]
	miR-22	*NLRP3*	[138]
	miR-195-5p	*TRIM14*	[139]
	miR-30a-5p	*FAP*	[140]
	miR-376c-3p	*HOXB7*	[141]
	miR-375	*PDGF-A*	[142]
	miR-320a	-	[143]
